# A method for simultaneous detection of small and long RNA biotypes by ribodepleted RNA-Seq

**DOI:** 10.1038/s41598-021-04209-4

**Published:** 2022-01-12

**Authors:** Nikita Potemkin, Sophie M. F. Cawood, Jackson Treece, Diane Guévremont, Christy J. Rand, Catriona McLean, Jo-Ann L. Stanton, Joanna M. Williams

**Affiliations:** 1 Department of Anatomy, School of Biomedical Sciences, University of Otago, P.O. Box 56, Dunedin, New Zealand; 2 Brain Health Research Centre, Brain Research New Zealand-Rangahau Roro Aotearoa, University of Otago, Dunedin, New Zealand; 3 Victorian Brain Bank, The Florey Institute of Neuroscience and Mental Health, Melbourne, VIC Australia; 4 Anatomical Pathology, The Alfred Hospital, Melbourne, VIC Australia

**Keywords:** Molecular biology, Neuroscience

## Abstract

RNA sequencing offers unprecedented access to the transcriptome. Key to this is the identification and quantification of many different species of RNA from the same sample at the same time. In this study we describe a novel protocol for simultaneous detection of coding and non-coding transcripts using modifications to the Ion Total RNA-Seq kit v2 protocol, with integration of QIASeq FastSelect rRNA removal kit. We report highly consistent sequencing libraries can be produced from both frozen high integrity mouse hippocampal tissue and the more challenging post-mortem human tissue. Removal of rRNA using FastSelect was extremely efficient, resulting in less than 1.5% rRNA content in the final library. We identified > 30,000 unique transcripts from all samples, including protein-coding genes and many species of non-coding RNA, in biologically-relevant proportions. Furthermore, the normalized sequencing read count for select genes significantly negatively correlated with Ct values from qRT-PCR analysis from the same samples. These results indicate that this protocol accurately and consistently identifies and quantifies a wide variety of transcripts simultaneously. The highly efficient rRNA depletion, coupled with minimized sample handling and without complicated and high-loss size selection protocols, makes this protocol useful to researchers wishing to investigate whole transcriptomes.

## Introduction

Over the last 50 years, it has gradually been accepted that previously-dubbed “junk DNA” plays vital biochemical roles in higher organisms. This DNA does not directly code for proteins yet makes up ~ 80% of the human genome. The gathering consensus is that by taking an holistic approach to the genome, that is not just examining protein-coding genes, it is possible gain a better understanding of the whole^1^. This concept extends to the investigation of the transcriptome by RNA sequencing (RNA-Seq), with this field already moving away from simply examining differential gene expression (DGE) of messenger RNA (mRNA), and towards investigation of other species of cellular RNA. Increasingly, non-coding RNA (ncRNA) have been shown to have numerous and varied biological roles. They have been implicated in disease aetiology and pathogenesis^2,3^, have high level of evolutionary conservation^4^ and stability^5–8^ and as such are attractive targets of research. Capturing these ncRNA species can, however, be more challenging than capturing mRNA. Many commercially available ncRNA sequencing kits exist, including but not limited to Illumina TruSeq Small RNA Sample kit, PerkinElmer NEXTFLEX Small RNA Library Prep Kit, and NEBNext Small RNA-Seq Kit. All of these methods rely on specifically isolating small RNA transcripts (usually < 160 bp) by size selection and excision of the region of interest from a solid matrix, followed by precipitating the RNA^9^. While this can allow deep sequencing of RNA within that size range, it is limited in two respects. First, a significant amount of information on the transcriptome is lost through RNA falling outside of the excised size range. Second, the precipitation of RNA from the gel will never be entirely efficient, resulting in unavoidable loss of material. It would be ideal, therefore, to develop a protocol that allows the researcher to simultaneously identify small non-coding RNA transcripts, as well as larger coding- and non-coding RNA, without material loss due to size selection.

The removal of ribosomal RNA (rRNA) from RNA samples is a crucial step in RNA-Seq methods. Ribosomal RNA is a considerable roadblock to the detection of other functionally relevant RNA species, as it makes up to 80–90% of total RNA in a cell (by mass)^10–12^. Current RNA-Seq protocols generally follow one of two rRNA removal methods—enrichment of polyadenylated (poly-A) RNA or depletion of ribosomal RNA (rRNA). Poly-A selection relies on the use of oligo dT primers to capture polyadenylated transcripts. This population is largely made up of mRNA, but does not capture all mRNA. Indeed, there is considerable evidence that a significant proportion of brain-derived mRNA is non-polyadenylated, further complicating the use of poly-A selection for investigating brain transcriptomes^13–17^. As a result, RNA-Seq data generated by positive capture of polyadenylated RNA do not represent information from non-polyadenylated transcripts, degraded RNA transcripts, and the vast majority of non-coding RNA species. By contrast, depleting total RNA samples of rRNA allows quantification of a more varied population of RNA species. rRNA depletion can be achieved by a variety of means, including dedicated rRNA removal kits. For example, Ribo-Zero Plus (Illumina), captures rRNA by hybridization to complimentary oligonucleotides (ONTs) coupled to magnetic beads that, when precipitated, remove the rRNA from the rest of the RNA. Another method relies on hybridizing rRNA to complementary DNA oligonucleotides. This is followed by RNaseH digestion of the RNA:DNA hybrids (NEBNext rRNA Depletion kit, Takara Bio RiboGone). Takara/Clontech SMARTer Stranded Total RNA-Seq kit also includes a proprietary method for rRNA removal that uses ZapR to degrade cDNA originating from rRNA. These methods show different rRNA depletion efficiency, depending on input RNA quality^18–21^ and furthermore, some variability in rRNA depletion efficiency has been reported between the implementation of the same protocol at different physical locations^18^.

Generally, the literature reports rRNA making up anywhere from 0.5 to 20% of final rRNA-depleted sequencing libraries^18–22^. With sequencing protocols usually generating in the vicinity of 20–30 million reads per sample, this can equate to 4–6 million reads mapping to rRNA. Better rRNA removal efficiency would result in those reads becoming available for mRNA and non-coding RNA sequence reads, of greater experimental interest to researchers. Furthermore, many of these techniques include multi-step protocols, often requiring precipitation steps (in the case of bead-based systems) and/or digestion or degradation steps. This often results in the loss of RNA material through purification, precipitation, or digestion. Thus the ideal rRNA removal technology would minimise workflow steps, sample handling, and reduce loss of material from precipitation or purification steps.

In a new development, Qiagen has released the QIAseq FastSelect rRNA removal kit, which utilizes complementary ONTs that bind to rRNA and prevent their reverse transcription to cDNA. The two main draws of this technology are its seamless integration into existing library preparation protocols (a single pipetting step and 14 min protocol), and the fact that it does not require any additional purification, precipitation, or enrichment steps, thereby minimizing sample loss. This considerable reduction in sample handling is key to accurate and efficient detection of especially low-abundance transcripts.

Another perceived hurdle in effective RNA-Seq is quality of the input RNA. While standardized methods exist for assessing RNA quality and the level of RNA degradation (most commonly RNA Integrity Number; RIN), there is no well-defined consensus on what constitutes a sample that is too degraded for RNA-Seq. Any cut-off for sample exclusion used in the literature is, therefore, almost entirely arbitrary^23,24^. This is not to say, however, that difficulties do not exist when performing RNA-Seq using samples of lower quality^25–27^. Firstly, as noted above, degraded RNA proscribes the use of Poly-A selection for rRNA removal, as the process of degradation renders poly-A selection inefficient, and introduces a strong 3’ gene end bias to sequenced reads^28,29^. Second, studies report that RNA samples of low quality (such as those obtained from post-mortem human tissue, in particular after a long post-mortem interval > 24 h) consistently show decreased proportions of mappable reads and a perceived reduction in sample complexity, with fewer highly-expressed genes and an abundance of low-expression genes^26^.

As part of an ongoing investigation into the transcriptome in Alzheimer’s disease, we have developed an end-to-end RNA-Seq workflow that addresses some of the shortcomings of currently available protocols, in particular for rRNA depletion, minimisation of sample loss, and handling of varying input RNA quality. We demonstrate that this protocol is capable of identifying and quantifying both coding and non-coding RNA (that is, all annotated types of RNA – small and long, coding and non-coding) simultaneously from both high-quality and degraded RNA samples.

## Results

### Modified library preparation protocol consistently produces high quality sequencing libraries

**Figure 1:**
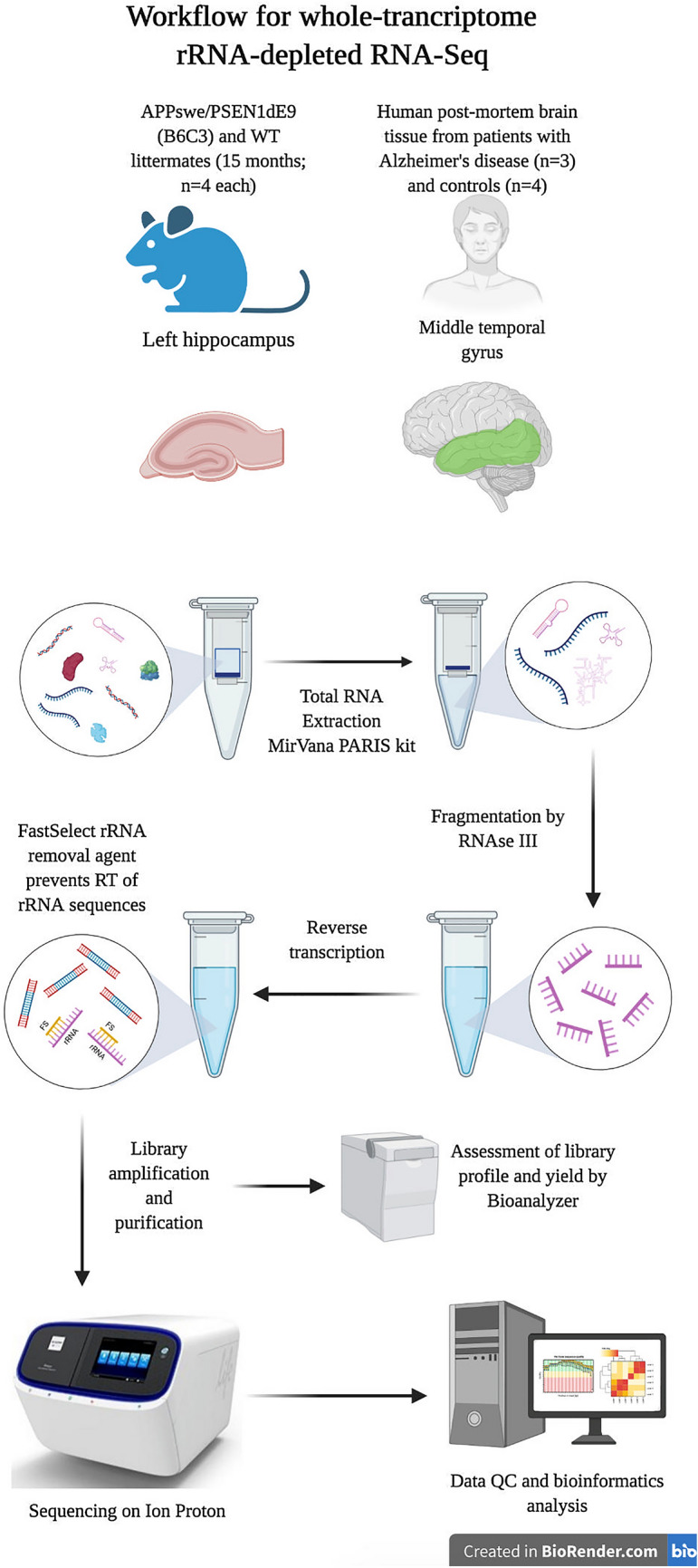
An overview of the protocol described here for simultaneous detection of coding and non-coding RNA by RNA-Seq. Created in BioRender.com.

**Figure 2:**
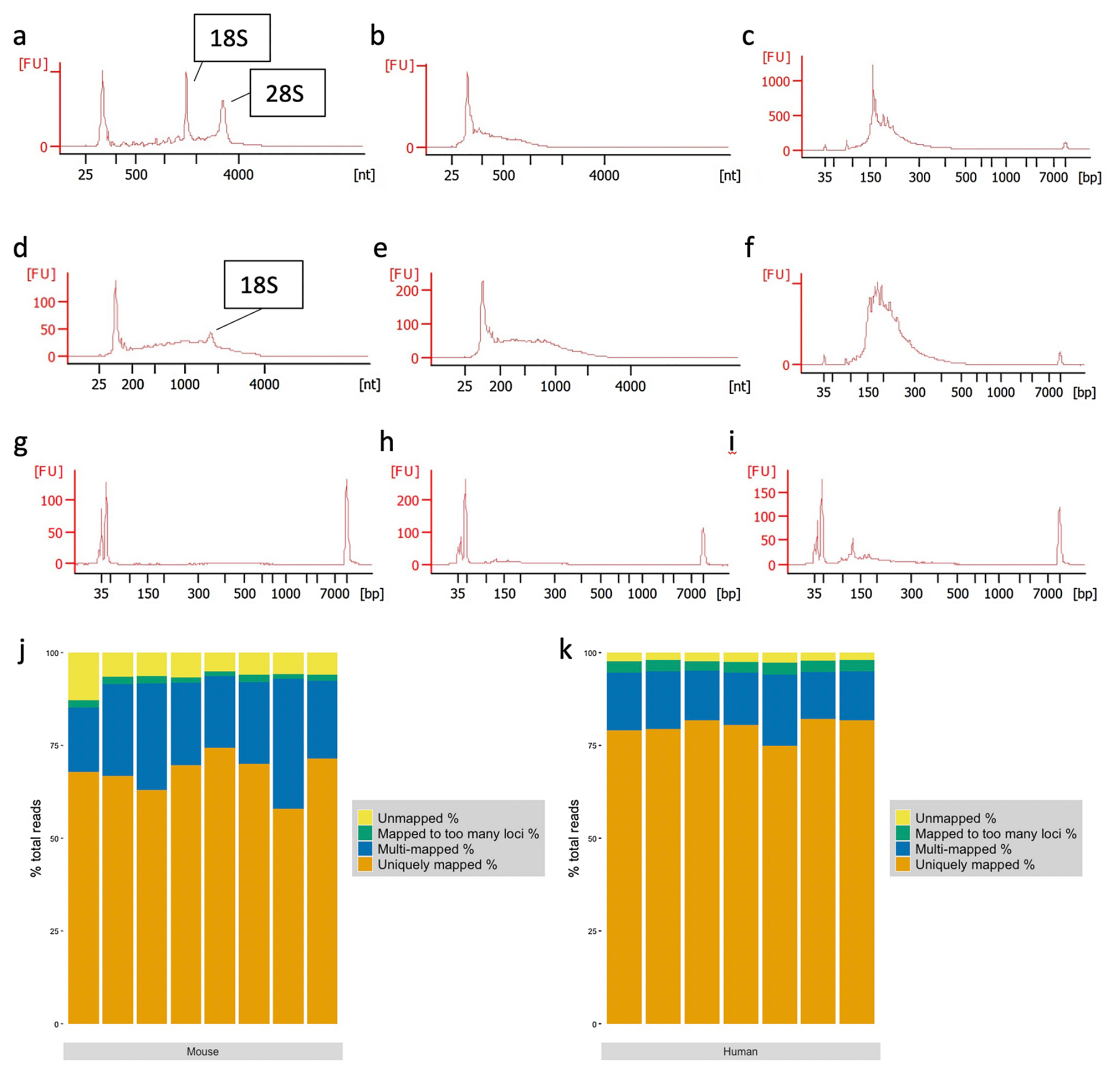
**(a–c)** Representative Bioanalyzer electropherograms of **(a)** total input RNA, **(b)** RNA after 8 min fragmentation by RNase III, and **(c)** final amplified cDNA library from mouse hippocampal RNA. **(d–f)** Representative Bioanalyzer electropherograms of **(d)** total input RNA, **(e)** RNA after 1 min fragmentation by RNase III, and **(f)** final amplified cDNA library from human MTG RNA. (**g–i**) Representative Bioanalyzer electropherograms of unamplified cDNA produced from adapter ligation for (**g**) 30 min at 30 °C (**h**) 60 min at 30 °C, and (**i**) 16 h at 16 °C. Yield of cDNA (size 50 to 1000 bp) markedly increases from 53 pg/μL (**g**) to 528 pg/μL (**h**) to 745.5 pg/μL (**i**) with increasing ligation time. (**j**) RNA reads mapped to the ENSEMBL *Mus musculus* GRCm38.95 annotated genome and (**k**) to the ENSEMBL *Homo sapiens* GRCh38.96 annotated genome. Uniquely mapped reads, multi-mapped reads, reads mapped to too many loci (> 10), and unmapped reads for each sample shown as a percentage of total trimmed and filtered reads.

To determine whether this protocol (overview shown in Fig. 1) can effectively be used to create whole transcriptome sequencing libraries from total RNA, we extracted RNA from fresh frozen tissue (hippocampal region of APP/PS1 and wild-type littermate control mouse brain) using the mirVANA Paris kit (Invitrogen) total RNA procedure. The extracted RNA was consistently highly concentrated and of high quality, as reported by the Agilent Bioanalyzer RNA Nano Chip (Table 1). The RIN was 9.1 ± 0.05 (mean ± standard deviation), with an average concentration of 174.6 ± 27.7 ng/μL. A260/280 ratios, as calculated by spectrometry, were > 2.12 (Table 1; 2.14 ± 0.01), strongly implying the lack of double-stranded DNA (dsDNA) contamination in the sample. RNA fragmentation by RNase III was optimised to 8 min to align with manufacturer’s recommendations. Representative electropherograms of total input RNA and fragmented RNA are shown in Fig. 2a,b. Total RNA (Fig. 2a) shows clear 18S and 28S rRNA peaks, while post-fragmentation these peaks become distributed with the overall RNA size distribution shifting downwards (Fig. 2b). The characteristic small RNA peak at ~ 100 nucleotides (nt) is also clearly seen and is retained post-fragmentation.

**Table 1:** RNA extraction from mouse tissue resulted in high-integrity, highly-concentrated total RNA.

Sample	RNA integrity number (RIN)	Concentration (ng/μL)	rRNA ratio [28S/18S]	Ratio A260/280
Sample 1	9	198	1.4	2.14
Sample 2	9.1	188	1.5	2.13
Sample 3	9.2	226	1.9	2.14
Sample 4	9.4	167	1.9	2.16
Sample 5	8.9	144	1.5	2.16
Sample 6	9	147	1.5	2.15
Sample 7	9.1	165	1.8	2.12
Sample 8	9.1	162	1.8	2.15
Average ± SD	9.1 ± 0.05	174.6 ± 27.7	1.66 ± 0.19	2.14 ± 0.01

Next, we assessed the remainder of the library preparation protocol, and a few modifications to the manufacturer’s protocol resulted in better outcomes overall. We adjusted the adapter ligation period to a 16-h (overnight) incubation at 16 °C, rather than the recommended 30 °C for 30 min. This markedly increased adapter ligation efficiency 15-fold (from 53 to 745.5 pg/μL; Fig. 2g–i). While this does increase the time required for library preparation, we found that this is outweighed by the increase in ligation efficiency.

The next key improvement is the removal of rRNA from the sample prior to reverse transcription, which was achieved by addition of the Qiagen FastSelect rRNA removal agent to the cDNA synthesis steps. Hybridization of the FastSelect ONTs was achieved by step-wise cooldown of the reaction mix from 65 to 25 °C, before addition of Superscript Enzyme Mix. This prevents rRNA from undergoing reverse transcription to cDNA. The efficiency of the rRNA removal step is explored further below. The final cDNA libraries showed size distributions in-line with manufacturer’s recommendations (up to 200-base fragments for the Ion Proton system), with < 50% of cDNA fragments falling under 160 base pairs (bp) (Fig. 2c).

The library preparation protocol described here resulted in sequencing libraries ranging from 25.5 to 159.2 nmol/L in concentration. Library and sequence run metrics are given in Table 2. The mean read length for each library ranged from 63 to 113 bp, with an average of 23,695,819 total raw reads. Removal of adapter sequences and filtering of low quality reads using the specifications described in the methods resulted in between 11,991,333 (sample 1) and 22,422,158 reads (sample 3). Between 73.36% and 83.77% of adapter-trimmed reads remained post-filtering.

**Table 2:** Library and sequencing metrics for mouse RNA samples.

Sample	Final library molarity (pmol/L)	Barcode ##	# raw reads	Mean read length (bp)	% duplicated sequences	# quality filtered reads	% post-filtering
Sample 1	25,963.90	1	17,487,705	63	62.58	11,991,333	73.36
Sample 2	25,459.80	2	20,424,675	86	63.85	14,329,078	80.61
Sample 3	71,161.70	5	31,493,446	92	50.62	22,422,158	77.9
Sample 4	39,349.50	6	27,138,089	93	52.07	18,407,710	72.99
Sample 5	109,683.40	3	21,131,492	96	59.54	16,486,584	83.77
Sample 6	55,390.00	4	24,032,524	113	54.54	18,236,544	80.07
Sample 7	90,893.50	7	25,463,277	90	46.71	19,414,166	80.23
Sample 8	159,175.50	8	22,395,347	103	49.77	17,274,480	80

Alignment to the reference genome resulted in between 58 and 75% uniquely mapped reads, 17.5% and 35% multi-mapped reads, 1.33% and 2% mapped to too many (> 10) loci, and 5–12% unmapped reads (Table 3; Fig. 2j). The numbers of uniquely mapped reads are consistently on the higher end of previously reported mapping statistics^30^.

**Table 3:** Read alignment statistics for mouse RNA samples.

Sample	Uniquely mapped reads	Uniquely mapped %	Multi-mapped reads	Multi-mapped %	Mapped to too many loci	Mapped to too many loci %	Unmapped %
Sample 1	8,129,518	67.79	2,098,856	17.5	221,679	1.85	12.86
Sample 2	10,658,465	74.38	2,760,489	19.26	191,067	1.33	5.02
Sample 3	14,989,536	66.85	5,545,647	24.73	427,075	1.9	6.51
Sample 4	12,882,689	69.99	4,051,474	22.01	370,911	2.01	5.99
Sample 5	9,556,157	57.96	5,757,276	34.92	234,205	1.42	5.7
Sample 6	11,483,874	62.97	5,237,205	28.72	364,546	2	6.31
Sample 7	13,532,973	69.71	4,321,515	22.26	259,941	1.34	6.69
Sample 8	12,333,236	71.4	3,644,129	21.1	267,752	1.55	5.96

### High-quality sequencing libraries even from fresh-frozen human post-mortem brain tissue

RNA derived from human post-mortem tissue can be challenging to extract intact, as variations in post-mortem interval, sample storage and transfer between locations may promote degradation of RNA within a sample. We therefore aimed to assess whether this protocol is capable of producing sequencing libraries from such samples. RNA extracted from human brain tissue was less intact, as determined by electropherography, with an average RIN of 2.3 ± 0.2, and of lower concentrations than mouse RNA from similar amount of tissue input (68.57  ± 15.77 ng/μL; Table 4, Fig. 2d). A260/280 ratios, as calculated by NanoDrop, all lay above 2 (Table 4; 2.09 ± 0.04), again suggesting that the samples did not contain dsDNA. Notably, however, the resulting libraries were comparable in concentration and size distribution to those resulting from high quality mouse RNA (122.4  ± 21.5  nmol/L; Table 5). Representative electropherograms of starting input RNA, fragmented RNA, and final libraries are shown in Fig. 4. While the input RNA (Fig. 2d) lacks the defined 18S/28S rRNA peaks seen in Fig. 2a, the 1 min fragmented RNA (Fig. 2e) electropherogram shows a very similar size distribution to 8 min fragmented mouse RNA (Fig. 2b). Again, the characteristic small RNA peak at ~ 100 nt is also clearly seen and is retained post-fragmentation. Similarly, the final library size distribution (Fig. 2f) is comparable to that seen in Fig. 2c. Library and sequencing statistics are shown in Table 5. The mean read length varied from 71 to 115 bp, with an average of 25,441,497 reads per sample. Quality filtering and adapter removal resulted in on average 15,855,518 reads per sample, leaving between 70 and 81% of reads post-processing.

**Table 4:** RNA extraction from human post-mortem tissue resulted in low-integrity total RNA.

Sample	RNA integrity number (RIN)	Concentration (ng/μL)	rRNA ratio (28 s/18 s)	Ratio A260/280
Patient 1	2.5	39.6	0.2	2.1
Patient 2	1.9	84.2	0	2.11
Patient 3	2.3	79.6	2.4	2.12
Patient 4	2.2	80.9	0	2.02
Patient 5	2.3	66.9	0.2	2.12
Patient 6	2.5	57.4	0	2.05
Patient 7	2.2	71.4	0	2.09
Average ± SD	2.27 ± 0.2	68.6 ± 15.8	0.4 ± 0.89	2.09 ± 0.04

**Table 5:** Library and sequencing statistics for human-derived RNA samples*.*

Sample	Final library molarity (pmol/L)	Barcode ##	# raw reads	Mean read length	% duplicated sequences	# quality filtered reads	% post-filtering
Patient 1	131,574.30	6	28,212,506	105	47.56	18,562,867	70.13
Patient 2	131,068.10	2	28,789,188	109	45.8	19,003,266	73.98
Patient 3	88,007.30	4	29,588,767	71	48.7	19,774,398	73
Patient 4	134,256.40	3	20,982,426	106	49.03	15,431,060	81.11
Patient 5	142,720.10	7	26,888,887	96	52.25	20,344,613	79.63
Patient 6	134,278.50	1	23,085,633	101	51.23	16,661,101	79.13
Patient 7	95,156.50	5	20,543,075	115	50.55	15,211,322	79.54

Alignment to the human reference genome uniquely mapped between 79 and 82% of reads, and multi-mapped between 12 and 15% of reads (Table 6; Fig. 2k). Only ~ 3% of reads were mapped to too many loci, and between 2 and 3% of reads were unmapped. The proportion of uniquely-mapped reads is consistent with previously described mapping statistics, though with a considerably lower percentage of unmapped reads^30,31^. We therefore demonstrate that the described protocol produces quality libraries from even fresh-frozen human post-mortem input RNA.

**Table 6:** Read alignment statistics for human-derived RNA samples.

Sample	Uniquely mapped reads	Uniquely mapped %	Multi-mapped reads	Multi-mapped %	Mapped to too many loci	Mapped to too many loci %	Unmapped %
Patient 1	14,689,409	79.13	2,869,622	15.46	554,660	2.99	2.42
Patient 2	15,312,048	80.58	2,673,472	14.07	523,535	2.75	2.6
Patient 3	14,815,243	74.92	3,796,504	19.2	628,450	3.18	2.7
Patient 4	12,681,322	82.18	1,948,225	12.63	465,695	3.02	2.17
Patient 5	16,171,475	79.49	3,154,060	15.5	627,631	3.08	1.92
Patient 6	13,619,025	81.74	2,197,338	13.19	505,216	3.03	2.04
Patient 7	12,427,195	81.7	2,033,866	13.37	398,154	2.62	2.32

### Ribosomal RNA removal by Qiagen FastSelect results in minimal rRNA content

To assess the effectiveness of Qiagen FastSelect rRNA removal agent, we used SeqMonk RNA-Seq QC to quantify the percentage of reads mapped to rRNA sequences in both the mouse and human samples. Ribosomal RNA content in RNA extracted from the mouse hippocampal tissue was between 0.23 and 2.58% (1.77 ± 0.91; n = 8; Table 7), and alignment to mitochondrial RNA (Mt-rRNA and Mt-tRNA) accounted for, on average, 0.25 ± 0.17% and 0.87 ± 0.49% respectively. Similarly, in the human RNA, the same protocol quantified rRNA content between 0.24 – 1.34% (0.45 ± 0.40; n = 7; Table 8), with mitochondrial rRNA and tRNA accounting for, on average, 0.28 ± 0.27 and 5.11 ± 3.23% respectively. As an average Ion PI Chip loaded with four samples returns ~ 25 million reads per sample, a total RNA library prep without rRNA depletion would result in ~ 22 million reads mapping to rRNA, whereas the protocol described here resulted in only ~ 100–200,000 reads mapped to rRNA. Compared to other techniques for rRNA removal from sequencing libraries, the technique described here performed consistently better that previously published methods, which range anywhere from 1 to 20% rRNA content^18–22^. Thus the considerable reduction in rRNA content achieved by our protocol frees up valuable sequencing resources.

**Table 7:** Percentage mouse RNA reads mapped to gene biotypes per sample, as annotated in the *Mus musculus* GRCm38.95, as well as tRNA annotations from UCSC Genome Browser, piRNA annotations from piRNABank.

Samples	lincRNA	snoRNA	snRNA	Pseudogenes	piRNA	miRNA	miscRNA	Antisense	Unknown	Protein coding	lncRNA	tRNA	rRNA	Mt-rRNA	Mt-tRNA
1	2.65	15.03	6.74	3.15	1.94	3.71	4.38	1.20	1.33	51.62	0.03	3.80	2.52	0.53	1.38
2	2.13	7.13	11.09	3.27	3.02	2.35	10.07	1.06	0.48	54.91	0.04	1.72	2.14	0.20	0.40
3	1.31	2.72	18.03	4.12	2.63	4.65	13.77	0.79	0.39	47.92	0.03	0.87	1.70	0.25	0.81
4	1.51	2.54	4.11	4.50	1.72	7.62	20.06	0.96	0.52	52.07	0.04	0.59	2.83	0.31	0.61
5	1.32	3.73	18.61	2.90	2.92	4.14	17.15	0.77	0.42	44.04	0.03	0.99	1.03	0.16	1.79
6	1.15	2.10	23.01	2.45	4.05	5.95	21.93	0.66	0.36	36.58	0.03	0.81	0.23	0.13	0.57
7	1.71	3.25	17.23	3.60	3.63	3.41	9.95	1.02	0.60	50.99	0.04	0.66	2.58	0.38	0.98
8	1.55	3.10	16.55	3.53	3.57	3.34	10.76	0.94	0.45	53.68	0.04	0.72	1.15	0.17	0.46

**Table 8:** Percentage human RNA reads mapped to gene biotypes per sample, as annotated in the *Homo sapiens* GRCh38.96, as well as tRNA annotations from UCSC Genome Browser, piRNA annotations from piRNABank.

Samples	lincRNA	snoRNA	snRNA	Pseudogenes	piRNA	miRNA	Antisense	Unknown	Protein coding	lncRNA	tRNA	rRNA	Mt-rRNA	Mt-tRNA
Patient 1	4.04	29.44	6.70	2.96	1.45	0.91	3.19	0.22	37.19	0.03	8.08	0.26	0.19	5.36
Patient 2	4.23	19.54	6.53	4.26	2.49	0.75	3.74	0.27	46.67	0.03	9.33	0.46	0.23	1.46
Patient 3	4.18	23.93	2.54	2.35	1.43	2.57	2.79	0.36	33.23	0.03	12.96	1.34	0.89	11.39
Patient 4	5.15	22.74	10.61	2.73	1.58	1.06	2.36	0.39	42.79	0.03	4.10	0.32	0.20	5.96
Patient 5	4.05	26.91	7.98	2.08	1.49	1.21	3.07	0.14	38.50	0.02	8.55	0.34	0.19	5.45
Patient 6	4.43	22.71	15.49	2.49	1.35	0.73	2.78	0.28	40.04	0.03	5.72	0.24	0.13	3.60
Patient 7	5.59	23.90	11.28	2.88	1.60	0.58	2.75	0.25	43.15	0.03	5.08	0.25	0.13	2.52

### This library preparation protocol and analysis pipeline identifies a variety of coding- and non-coding-RNA in biologically-relevant proportions

To achieve an estimate of the ability of this workflow to identify transcripts of interest, we performed bioinformatic analysis to determine (a) how many different transcripts can be identified from the RNA-Seq data and (b) what kind of transcripts can be identified.

**Figure 3:**
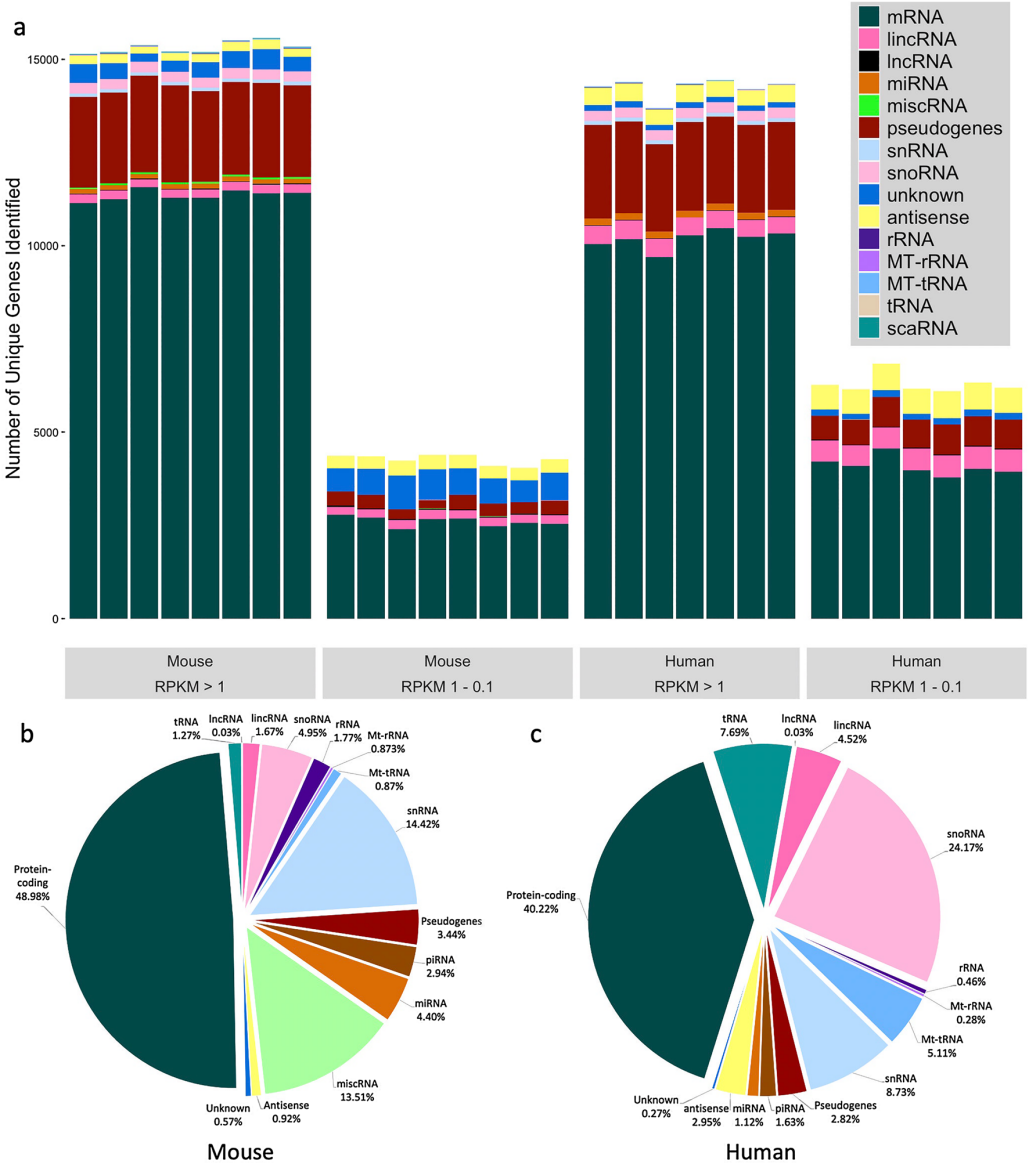
Breakdown of number of genes identified by RNA biotype. (**a**) Number of genes detected at > 1 Reads per Kilobase Million (RPKM) and > 0.1 RPKM for each mouse sample and human sample, divided by gene biotype. RPKM here was used as a proxy for normalized expression. (**b,c**) Percentage RNA reads mapped to gene biotypes for (**b**) mouse and (**c**) human samples, averaged across samples.

We calculated normalized Reads Per Kilobase Million (RPKM) for mouse and human RNA samples to normalise the number of unique transcripts detected for sequencing depth and gene length. RPKM was chosen here over the more commonly used transcripts per million reads (TPM) to allow direct comparison to other published reports of genes detected^18^. Mouse samples identified > 15,000 unique transcripts expressed at greater than one RPKM and an additional 4000 expressed at greater than 0.1 RPKM (Fig. 3a). For the human samples, a similar number of transcripts were found at > 1 RPKM, with more than ~ 6000 additional unique transcripts at > 0.1 RPKM (Fig. 3a). The number of genes detected at RPKM > 1 were comparable to that reported by other rRNA-depleted RNA-Seq protocols^18^. While the number and type of genes identified at RPKM > 1 were very similar between mouse and human samples, human samples showed a greater number of lowly-expressed genes (Fig. 3a). These findings are in stark contrast to some of the reported difficulties in RNA-Seq using low-quality input RNA—notably decreased proportions of mappable reads and reduction in sample complexity^26^. In fact, our data suggest that this protocol results in proportions of successfully mapped reads and levels of gene expression comparable to high-quality, undegraded RNA samples.

**Figure 4:**
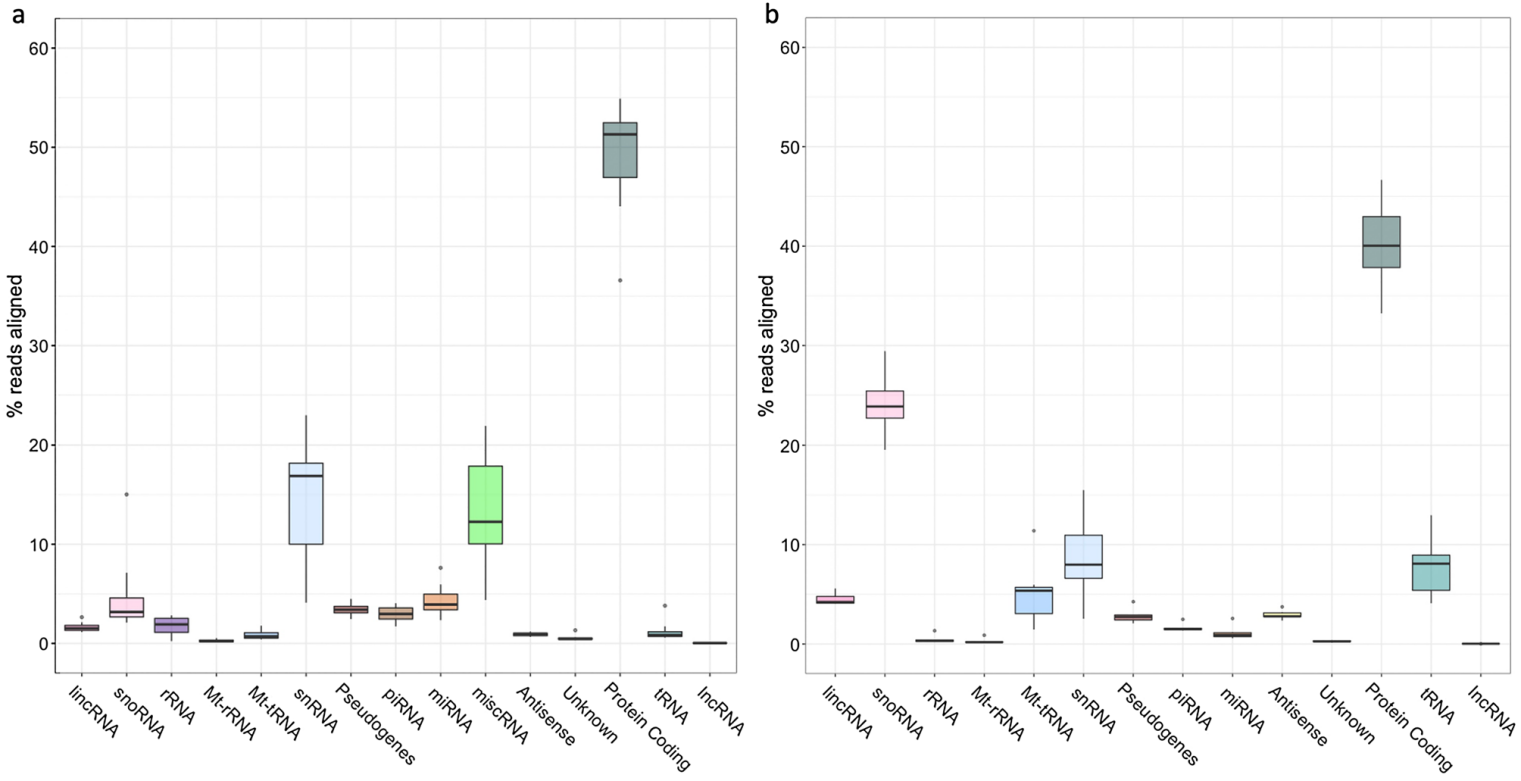
Box and whisker plot showing the range of percentages of reads mapped to gene biotypes for (**a**) mouse and (**b**) human samples. The majority of RNA species in both samples show very small ranges, while some (notably snRNA and snoRNA) are more variable between samples.

Breakdown of read alignment by transcript biotype (as annotated in each reference genome—*Mus musculus* GRCm38.100 and *Homo sapiens* GRCh38.96—as well as piRNA and tRNA from piRNABank and UCSC Genome Browser respectively) is shown in Table 7 and 8. The average percentage content by gene biotype is shown in Fig. 3b,c. The largest number of reads mapped to protein-coding mRNA (Mouse—48.86% ± 6.02; human—40.22% ± 4.43). There were numerous alignments to various species of ncRNA, including miRNA, piRNA, snRNA, snoRNA, lincRNA, and pseudogenes. With the removal of rRNA from the prepared libraries, proportions of ncRNA correspond approximately to reported cellular RNA contents^32^. Additionally, the method described here, when compared to data obtained from more conventional library construction methods for both mouse^33,34^ and human samples^35^, results in not only a greater number of total unique genes identified, but also a greater proportion of ncRNA biotypes compared to protein-coding mRNA (Supplementary Figs. 1 and 2). Many species of non-coding RNA show very narrow variance between samples (Fig. 4), while others varied significantly. In particular, small nuclear RNA (snRNA) expression was highly variable, ranging from 4 to 23% in mouse RNA (Fig. 4a), and 2.5 to 15.5% in human RNA (Fig. 4b). Despite their frequent use as reference genes for qRT-PCR and gene array experiments, individual variability in snRNA expression has been reported previously^36,37^, and these observations are supported by the data presented here.

This method was able to identify genes that have previously been reported to be differentially-expressed in both the APP/PS1 transgenic mouse model of AD, and in AD patients. Notably, among the top 20 DE genes in APP/PS1 mice, both *App* and *Psen1* were highly altered (log fold change 1.22 and 0.85 respectively, both p < 0.0001; Supplementary Table S1). Similarly in human post-mortem AD samples, we identified key differentially-expressed genes such as *GFAP* (log2 FC 1.48, p < 0.0001), *SERPING1* (log2 FC 1.02, p < 0.05), *KRT5* (log2 FC -3.52, p < 0.0001), and *NEUROD6* (log2 FC -1.23, p < 0.05), all genes that have been reported to be altered in AD^38^ (Supplementary Table S2). We also identified differentially-expressed miRNA that had previously been discovered in APP/PS1 mice^39,40^ (miR-26a-5p, log2 FC 0.58, p < 0.05; miR-7b-5p, log2 FC -0.55, p < 0.05; Supplementary Table S3) and human AD patients^41,42^ (miR-129-5p, log2 FC -1.14, p < 0.05; miR-151b, log2 FC -1.28, p < 0.05; Supplementary Table S4). As is often recommended for RNA-Seq experiments, further investigation and validation of differentially-expressed transcripts by, for example, quantitative RT-PCR would address concerns with regards to quantification.

### Select gene sequencing reads significantly correlate with cycle threshold (Ct) values obtained by quantitative RT-PCR

**Figure 5:**
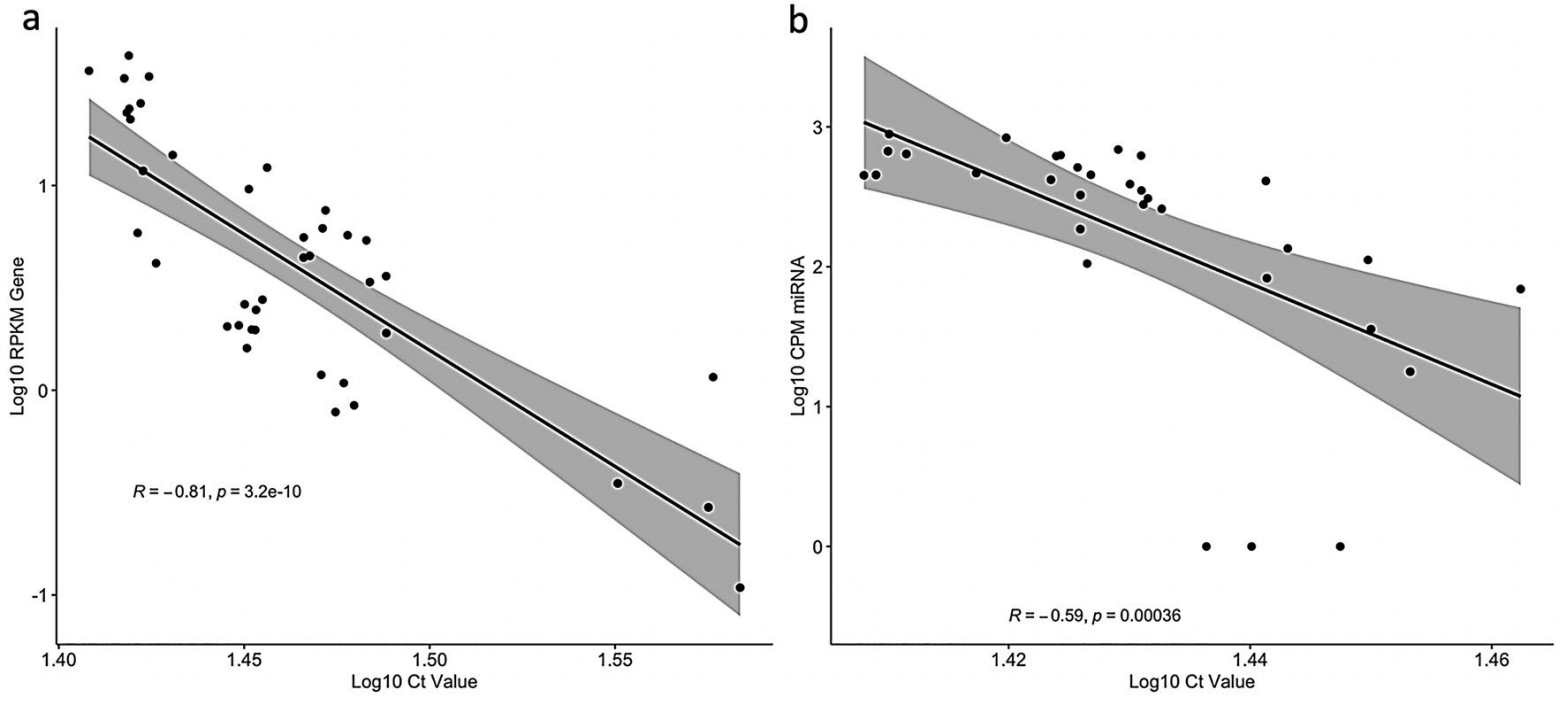
Correlation between Ct values from qPCR and normalized sequencing reads mapped to select genes (**a**) and miRNA (**b**). Gene sequencing reads were normalized for library size and gene length using RPKM, while miRNA reads were normalized for library size using CPM. Both sets of data passed Shapiro–Wilk tests of normality (p > 0.05). The linear regression line, confidence interval, Pearson’s correlation coefficient, and significance value are indicated.

In order to ascertain to what extent sequencing reads obtained from this protocol can be representative of the actual number of RNA molecules in the sample, we performed qRT-PCR analysis of selected genes and miRNA from the mouse samples. We then determined the correlation coefficient (Pearson’s Product-Moment Correlation) of Ct values against normalized read counts (RPKM for mRNA; CPM for miRNA), in order to control for library size and gene length (Fig. 5). Counts per Million reads (CPM) were used for the miRNA for simplicity, due to the lack of variability in mature miRNA gene length. We found that both mRNA (*Hprt*, *Trem2*, *Tyrobp*, *c-Fos*, and *Cst7*; R = -0.81, p = 3.2e−10; Fig. 5a) and miRNA (miR-129-1-3p, miR-34a-5p, miR-34c-5p, miR-210-3p; R = −0.59, p = 0.00036; Fig. 5b) showed significant negative correlation between Ct values and normalized sequencing reads. This strongly suggests that data obtained from this sequencing method results in read counts that are largely representative of actual RNA content and lends credence to differential gene expression analyses performed with these data.

## Discussion

RNA sequencing is an ever-evolving technique that offers unique insights into the transcriptome. Current protocols often require the researcher to choose between investigating mRNA (by poly-A selection) or small RNA (by size selection). Either one of these alone, while offering depth of sequencing, misses out on a great deal of information from excluded transcripts. Here, we report a significant advancement in RNA-Seq methodology, a novel method to investigate the whole transcriptome, from the same sample at the same time, using ribosomal-depleted RNA. This approach takes advantage of several existing commercially available kits, with some important alterations to manufacturer’s protocols. This altered workflow resulted in high quality sequencing libraries from input RNA samples of a variety of quality, from both mouse and human tissue. Low quality input RNA had no negative effect on the final library quality. Qiagen FastSelect rRNA removal agent integrated seamlessly into the existing Ion Total RNA-Seq kit v2 library prep protocol and resulted in highly effective depletion of rRNA from the final libraries, even from degraded samples, which is often a drawback of other rRNA removal techniques. A high number of genes were identified in the RNA-Seq data, including transcripts often overlooked by more targeted RNA-Seq protocols (refer to Fig. 3b,c). The majority of reads mapped to species of non-coding RNA, and most of these were also highly consistent between samples within each species. Furthermore, sequencing reads (normalized to library size and gene length) correlate significantly with Ct values from qRT-PCR quantitation (which allows for precise quantification of RNA content), suggesting that read counts obtained from this RNA-Seq protocol can be used to infer quantitative gene expression. One important caveat to consider, however, is that PCR amplification is known to favour smaller fragments over larger ones^43^. As such, it is possible (even likely) that transcripts associated with small non-coding RNA are overrepresented in our final sequencing libraries. However, this effect is likely consistent across samples and libraries, and would certainly not affect the identification of unique transcripts.

A similar protocol (fragmented, ribodepleted TGIRT-Seq) has been previously reported^44–46^, that also aimed to simultaneously sequence coding and non-coding RNA. While it has seen some limited implementation since^47,48^, it is yet to be widely accepted. There are a few factors where we believe improvements can be made. First, the use of QIASeq FastSelect for rRNA depletion appears to perform better than the RiboZero Gold used by Boivin and colleagues. While we did not compare these two methods directly, the comparison can be inferred through the literature. RiboZero Gold is no longer available to purchase and has been replaced by RiboZero Plus. However, there are no published data available to compare rRNA removal efficiency of RiboZero Plus. Crucially, rRNA-removal by FastSelect requires significantly less sample handling than RiboZero Gold, and does not require additional bead purification, preventing sample loss. Second, the protocol described here appears to give more representative non-coding RNA reads—in particular with regards to miRNA and piRNA—as compared to estimated abundances reported in literature^32,44^.

We recognise the variety of bioinformatics tools available for the analysis of RNA-Seq data. These include *bwa*, *Bowtie2*, *TopHat2*, *cufflinks*, and *HISAT2* for read mapping/alignment, software such as *kallisto* or *salmon* for direct quantification, differential gene expression tools such as *DESeq2*, *Cuffdiff*, and *limma*, and integrated pipelines such as *exceRpt*. Several studies have aimed to evaluate the relative sensitivity and accuracy of these analysis tools and pipelines, but for the most part, no one tool or pipeline consistently performed better than the others^49–53^. An in-depth discussion of these tools is outside the purview of this report.

The ability to capture sequencing reads from a wide variety of RNA species, coding and non-coding, is valuable to investigate many aspects of the transcriptome. In our research into Alzheimer’s disease, the ability to take a snapshot of the RNA environment allows us a unique insight into AD pathology, both in the APP/PS1 mouse model and in human post-mortem brain tissue. In particular, since non-coding RNA is being increasingly implicated in the aetiology and pathogenesis of AD^54–56^, knowing the changes that occur in the disease state can aid in understanding the disease, developing diagnostic tools, and hopefully developing new treatments. Since non-coding RNA are such a ubiquitous aspect of cellular function, the same approach, and therefore this method, can be applied to a variety of diseases and research areas.

Altogether, we believe this workflow may be useful to researchers wishing to investigate the whole transcriptome simultaneously, with effective rRNA depletion, and without complicated and high-loss size selection protocols commonly used for small RNA-Seq, or poly-A selection for mRNA-Seq.

## Materials and methods

### Animal studies

All animal use was compliant with the New Zealand Animal Welfare Act 1991 and performed under guidelines and approval of the University of Otago Animal Ethics Committee (approval number DET09/15). The reporting in this manuscript follows the recommendations in the Animal Research: Reporting on In Vivo Experiments (ARRIVE) guidelines^57^. In this study we utilised a double transgenic model of Alzheimer’s disease (APPswe/PS1dE9, B6C3 background, referred to as APP/PS1) originally sourced from The Jackson Laboratory (https://www.jax.org/strain/004462) and maintained as a colony at the University of Otago breeding facility. Mice were housed under specific pathogen-free conditions in a day-night controlled light cycle, with food and water access ad libitum. Animals underwent no additional procedures prior to their stated use. All mice were genotyped for the presence of human exon-9-deleted variant PSEN1, which co-segregates with the APPswe gene, as previously described^58^. Male transgenic (tg) and wild-type (wt) littermates at 15 months old (*n* = 4 per group) were anaesthetised with sodium pentobarbitol and the brains removed into ice cold artificial cerebrospinal fluid solution (aCSF; in mM: 124 NaCl, 3.2 KCl, 1.25 NaH_2_PO_4_, 26 NaHCO_3_, 2.5 CaCl_2_, 1.3 MgCl_2_, 10 d-glucose). The left hippocampus was dissected and snap-frozen on dry ice. All samples were stored at -80 °C until used. RNA extracted from mouse hippocampi is henceforth referred to by the identifier “Sample #”.

### Human studies

Use of human tissue was approved by and compliant with the guidelines of the New Zealand Health and Disability Ethics Committee (14/STH/20/AM07), the Human Research Ethics Committee of The University of Melbourne (1545740—for patient tissue banking and consent), and the Victorian Institute of Forensic Medicine (EC 18–2019). Informed consent was obtained from all donors. Post-mortem middle temporal gyrus (MTG) samples were received from the Victorian Brain Bank (VBB). Age-matched healthy control brains (n = 4; 2 male, 2 female; age 80.5 ± 8.8) were defined as free from Alzheimer’s Disease (AD) lesions with numbers of plaques and tangles below the cut-off values for a neuropathological diagnosis of AD (NIA Reagan criteria). No other neurological diseases were present. Alzheimer’s disease brains (n = 3; 3 female; age 76.5 ± 7.7) met the standard criteria for Alzheimer’s disease neuropathological diagnosis. There were no significant differences between the ages of the two groups (two-tailed t-test, p = 0.55). Patient sex was self-reported. All samples were stored at −80 °C until used. RNA extracted from human MTG samples is henceforth referred to by the identifier “Patient #”. Patient demographics and case information are available in Table 9.

**Table 9:** Case information of source of human post-mortem MTG tissue.

Sample	Age	Sex	PMI	Diagnosis	pH	Cause of death	Medication	Thal phase	Braak stage	CERAD score
Patient 1	78.9	Female	19.5	AD	6.46	Not available	Not indicated	4 or 5	V or VI	Moderate
Patient 2	82.7	Female	28.5	Control	6.44	Cardiac tamponade	None	0	None	None
Patient 3	78.8	Female	19	Control	6.27	Aortic dissection	None	0	None	None
Patient 4	69.6	Male	71	Control	6.65	Ischaemic heart disease	None	0	None	None
Patient 5	82.7	Female	9	AD	6.45	Pneumonia	Not indicated	3	V or VI	Frequent
Patient 6	90.8	Male	32.5	Control	6.47	Pneumonia	None	0	None	None
Patient 7	67.8	Female	21	AD	6.74	Alzheimer's disease	Haloperidol	4 or 5	V or VI	Frequent

Shows age at death, self-reported gender, post-mortem interval (PMI; hours), diagnosis, pH of tissue, immediate cause of death, medication taken (if any), and post-mortem pathology.

### RNA extraction

Total RNA was extracted from previously-frozen tissue using the mirVana™ PARIS™ RNA isolation kit (Invitrogen; Cat #AM1556), according to the manufacturer’s instructions. The concentration and purity were determined by both spectrophotometry (A260, A260/280 respectively; NanoDrop 1000 Spectrophotometer; NanoDrop Technologies, Waltham, MA) and capillary electrophoresis (RNA Integrity Number [RIN], RNA 6000 Nano chip, Cat #5067-1511; Agilent Bioanalyzer 2100, Agilent Technologies).

### Library preparation

Except where explicitly stated, all samples, regardless of species or group of origin, were treated identically. Sequencing libraries were prepared for Ion Proton using the Ion Total RNA-Seq kit v2 (Life Technologies; Cat #4479789) largely following manufacturer’s instructions. Total RNA (500 ng) was used as input to the Ion Total RNA-Seq kit v2 (356 ng input was used instead of 500 ng for Patient 1 due to low RNA yield), to which was added 1 μL of 1:100 ERCC Spike-In Mix 1 (Invitrogen; Cat #4456740), commonly employed to control for cross-sample variation in library preparation. RNA fragmentation by RNase III was performed at 37 °C. The fragmentation time was optimised to 8 min for the mouse RNA, and 1 min for the human RNA. This will vary depending on quality and integrity of the input RNA material. The resulting fragmented RNA was purified using the Magnetic Bead Cleanup Module (Life Technologies; Cat #4475486), and purified RNA eluted in 13 μL nuclease-free water.

Ligation of Ion adapters (Ion RNA-Seq Primer Set v2; Cat #4479789) was performed using 3 μL of the eluted purified fragmented RNA, added to 2 μL Ion Adapter Mix v2 and 3 μL Hybridization solution, and incubated in a thermal cycler at 65 °C for 10 min followed by 30 °C for 5 min. To this hybridization reaction was added 10 μL 2× Ligation Buffer and 2 μL Ligation Enzyme Mix, and incubated at 16 °C for 16 h in a thermal cycler. Following ligation, reverse transcription (RT) and rRNA removal was performed simultaneously as follows. RT master mix was prepared on ice (per sample; 1 μL nuclease-free water, 4 μL 10× RT buffer, 2 μL 2.5 mM dNTP Mix, 8 μL ion RT Primer v2, 1 μL QIAseq FastSelect rRNA removal agent). QIAseq FastSelect rRNA removal agent (Qiagen, Cat #334386) consists of ONTs complementary to ribosomal RNA sequences. These ONTs, when bound to rRNA sequences, prevent reverse transcription. The master mix was added to the ligation reaction, and incubated at 70 °C for 10 min, followed by a step-wise cooldown (2 min at 65 °C, 2 min at 60 °C, 2 min at 55 °C, 5 min at 37 °C, 5 min at 25 °C, hold at 4 °C). This step is necessary for the oligonucleotides in the FastSelect rRNA removal agent to bind rRNA fragments and prevent reverse transcription. Finally, 4 μL 10× Superscript Enzyme Mix was added to each reaction and the reactions incubated at 42 °C for 30 min.

The resulting cDNA was purified using the Magnetic Bead Cleanup Kit, and eluted in 12 μL nuclease-free water. In order to amplify the cDNA, 6 μL of this elution was added to a master mix of 45 μL Platinum PCR Supermix, 1 μL Ion Xpress 3’ Barcode Primer, and 1 μL Ion Xpress RNA Barcode BC## (Life Technologies; Cat #4475485). This mixture was amplified in a thermal cycler for 14 cycles (Hold 2 min 94 °C; Cycle 2× [94 °C 30 s; 50 °C 30 s; 68 °C 30 s]; Cycle 14× [94 °C 30 s; 62 °C 30 s; 68 °C 30 s]; Hold 5 min 68 °C). The amplified cDNA was purified again using the Magnetic Bead Cleanup Kit, and analysed by capillary electrophoresis (High Sensitivity DNA chip, Cat #5067-4626; Agilent Bioanalyzer 2100).

### Sequencing on ion proton platform

The prepared sequencing libraries were diluted to equimolar concentrations of 100 pmol/L for pooling. Emulsion PCR was performed with the Ion OneTouch™ 2 system (Invitrogen) using the Ion PI™ Hi-Q™ OT2 200 kit (Invitrogen; Cat #A26434) according to the manufacturer’s instructions. The four pairs of mouse samples (tg and wt) were processed simultaneously end-to-end, as were the seven human MTG samples. Libraries were sequenced on Ion PI™ v3 chips (Invitrogen; Cat #A26770), prepared using the Ion PI™ HiQ™ Seq 200 kit (Invitrogen; Cat #A26433, A26772). The mouse samples of two pools of mixed barcoded libraries were sequenced on two Ion PI v3 chips (2 wt and 2 tg per chip), avoiding the use of all sequential barcodes on the same chip. Similarly, human MTG libraries were sequenced in two pools of mixed barcoded libraries—one contained a pool of four samples (2 AD and 2 control), the other a pool of three samples (1 AD and 2 control).

### Quantitative reverse transcription polymerase chain reaction (qRT-PCR)

For gene expression qRT-PCR, using 350 ng starting total RNA input from mouse samples, cDNA was generated using SuperScript IV First Strand Synthesis System (Invitrogen; Cat #18091050) per manufacturer’s instructions, utilizing priming by random hexamers. Of this cDNA, a 1:25 dilution was used for the qRT-PCR reaction, which was performed using TaqMan Gene Expression Master Mix (Applied Biosystems, Cat #4369016), with the following TaqMan gene primers: Mouse *Hprt* (Assay ID: 03024075_m1), *Cst7* (00438351_m1), *Tyrobp* (00449152_m1), *c-Fos* (00487425_m1), *Trem2* (04209424_m1). The reactions were amplified on the Applied Biosystems ViiA 7 system as follows: Hold 50 °C 2 min, hold 95 °C 10 min, and 40 cycles at 95 °C for 15 s and 60 °C for 1 min.

For miRNA, 10 ng of total RNA from mouse samples was used. cDNA was generated using the TaqMan MicroRNA Reverse Transcription Kit (Applied Biosystems, Cat #4366596) according to manufacturer’s instructions. The qRT-PCR reactions were prepared using TaqMan Universal PCR Master Mix (Applied Biosystems, Cat #4304437), with the following TaqMan miRNA primers: miR-34a-5p (Assay ID: 000426), miR-34c-5p (000428), miR-129–1-3p (002298), miR-210-3p (000512). The reactions were amplified on a Applied Biosystems ViiA 7 system as follows: Hold 50 °C 2 min, hold 95 °C 10 min, and 40 cycles at 95 °C for 15 s and 60 °C for 1 min. Genes and miRNA were chosen as a mix of housekeeping genes, genes that are known to be changed from the literature, and some genes of interest whose expression in APP/PS1 mice is unknown.

MicroRNA and mRNA qRT-PCR data were processed separately to account for differing input RNA amounts, and in each case, raw Ct values were used for analysis.

### Data analysis

Data from each barcoded library were separated into different data files automatically on the Ion Torrent Suite version 5.4 (life Technologies, USA). The Ion Torrent Suite was also used for analysis of ERCC Spike-In controls. Sequence read quality was evaluated using FastQC v0.11.5 (https://www.bioinformatics.babraham.ac.uk/projects/fastqc/)^59^. Adapter sequences were trimmed from reads using AdapterRemoval v2.1.7 (https://github.com/MikkelSchubert/adapterremoval/)^60^. Reads were then trimmed for quality using Trimmomatic v0.38 (http://www.usadellab.org/cms/?page=trimmomatic)^61^ using a 5-base sliding window, cutting when the average quality per base drops below 20, and dropping reads less than 17 bases long.

Mouse RNA reads were aligned to the *Mus musculus* GRCm38.95 reference genome (available on the Ensembl website: http://www.ensembl.org/info/data/ftp/index.html) and human RNA reads to the *Homo sapiens* GRCh38.96 reference genome using STAR v2.5.4b (https://github.com/alexdobin/STAR)^62^. Reference .gtf files for RNA biotypes (protein-coding, pseudogenes, snRNA, snoRNA, unknown [TEC], Mt-RNA, lncRNA, lincRNA, antisense) were extracted from the *Mus musculus* GRCm38.95 and *Homo sapiens* GRCh38.96 annotation files using the grep command.

MicroRNA (miRNA) were quantified from aligned counts using miRDeep2 v0.1.2 (https://github.com/rajewsky-lab/mirdeep2)^63^.

Piwi-interacting RNA (piRNA) sequences were obtained from piRNABank (http://pirnabank.ibab.ac.in)^64^.

Sequences for tRNA were obtained from the UCSC Genome Browser (http://genome.ucsc.edu).

Data were analysed using R version 4.0.2 in RStudio v1.3.959. The package *Rsamtools*^65^ was used to convert STAR output .sam files to .bam files. The *featureCounts* function from the package *Rsubread*^66^ was used to generate counts tables using aforementioned .gtf annotation files. The package *edgeR*^67^ was used to generate a DGEList object from feature counts, filtered for lowly-expressed genes by the function *filterByExpr*, and the function *exactTest* used to perform differential expression analysis.

The following packages were used to aid data handling and visualisation: *ggplot2*^68^, *ggpubr*^69^, *tidyverse*^70^. Additional statistics (regression/correlation) were also performed using R. Additional analysis and data visualisation performed using SeqMonk v1.45.1 (https://www.bioinformatics.babraham.ac.uk/projects/seqmonk/).

## Supplementary Information

Supplementary Information.

## Data Availability

The data discussed in this publication have been deposited in NCBI's Gene Expression Omnibus^71^ and are accessible through GEO Series accession numbers: GSE163877 (https://www.ncbi.nlm.nih.gov/geo/query/acc.cgi?acc=GSE163877) and GSE163878 (https://www.ncbi.nlm.nih.gov/geo/query/acc.cgi?acc=GSE163878).
