# Supplementary material for: A method for simultaneous detection of small and long RNA biotypes by ribodepleted RNA-Seq
Source: Sci Rep. 2022 Jan 12;12:621. doi: 10.1038/s41598-021-04209-4 (PMC8755727; doi:10.1038/s41598-021-04209-4)
Supplement: Supplementary file 1 — Supplementary Information. [file 41598_2021_4209_MOESM1_ESM.docx]

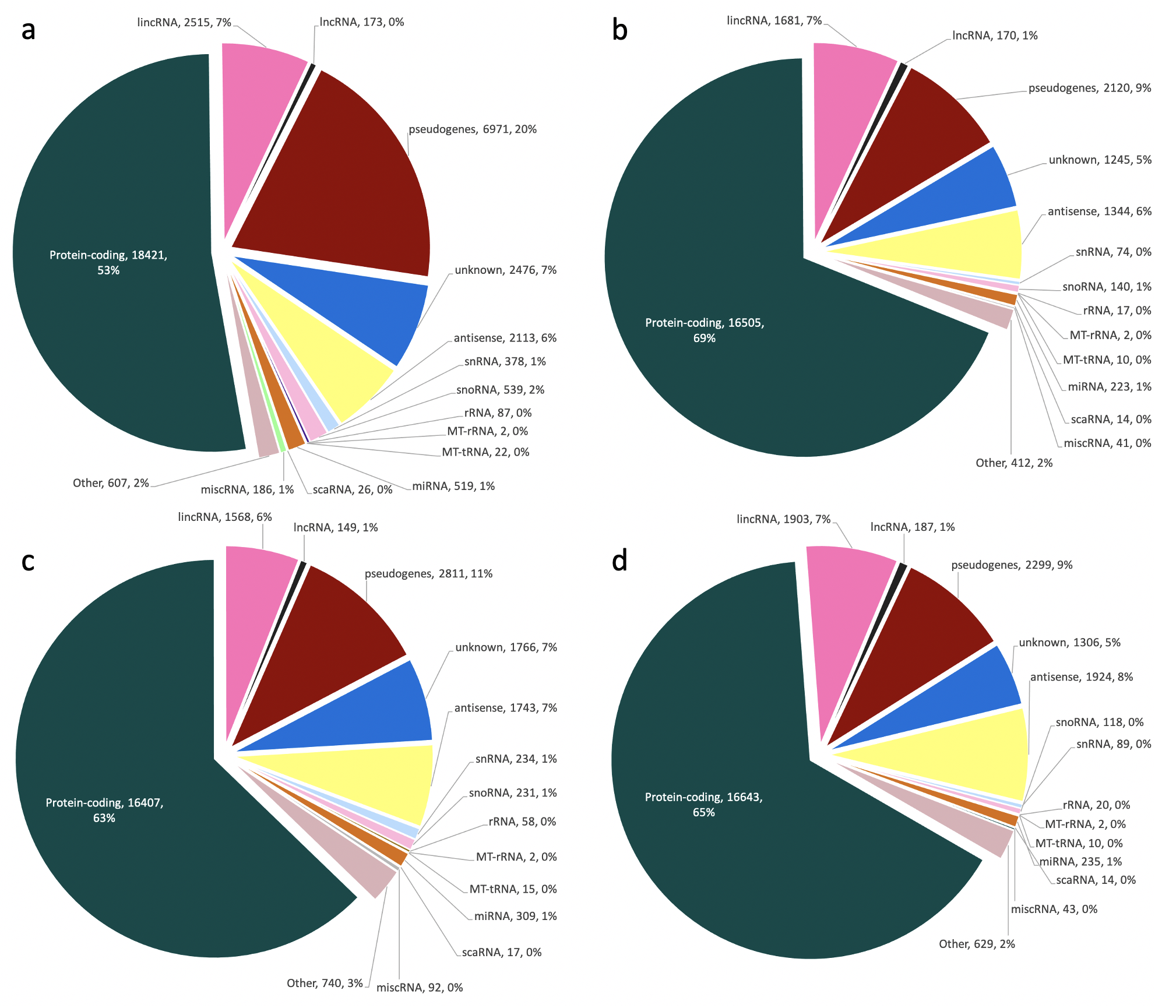


Supplementary Figure S1: Pie charts showing numbers and proportions of RNA biotypes identified using different ribodepleted library construction methods APP/PS1 and WT control mouse brain tissue (additional data obtained from GEO). (a) Number of unique genes by biotype identified in the data described here. (b) Number of unique genes by biotype identified in the data of Li and colleagues ^34^ (GEO accession GSE174314). (c) Number of unique genes by biotype identified in the data of Pan and colleagues ^33^ (GEO accession GSE137028). (d) Number of unique genes by biotype identified in the data of Gaunt and colleagues (data accessible at NCBI GEO database, accession GSE186710).


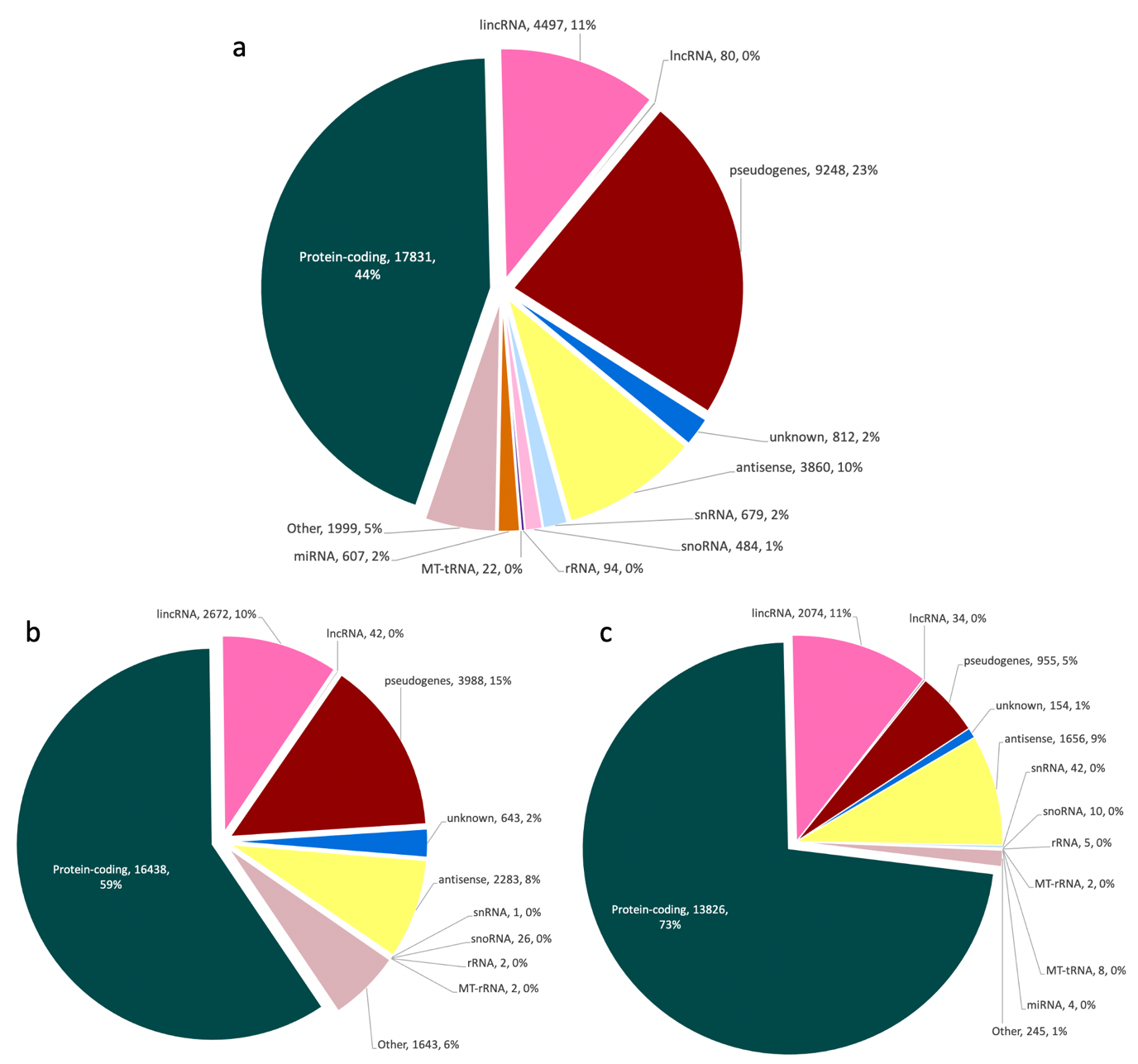


Supplementary Figure S2: Pie charts showing numbers and proportions of RNA biotypes indentified using different ribodepleted library construction methods using human post-mortem brain tissue from patients with Alzheimer’s disease and controls (additional data obtained from GEO). (a) Number of unique genes by biotype identified in the data described here. (b) Number of unique genes by biotype identified in the data of Wang and colleagues (GEO accession GSE184942). (c) Number of unique genes by biotype identified in the data of Gerrits and colleagues ^35^ (GEO accession GSE148822).

Supplementary Table S1: Top 20 genes differentially expressed between hippocampal tissue from APP/PS1 transgenic mice and wild-type littermates.

| Ensembl ID | Gene symbol | Log2 FC | Log2 CPM | P-Value |
| --- | --- | --- | --- | --- |
| ENSMUSG00000081229 | *Lamr1-ps1* | 6.729103 | 1.044629 | 3.38E-19 |
| ENSMUSG00000046805 | *Mpeg1* | 1.990667 | 4.700738 | 2.06E-17 |
| ENSMUSG00000030789 | *Itgax* | 3.608 | 1.839671 | 8.00E-13 |
| ENSMUSG00000068129 | *Cst7* | 3.439047 | 1.547066 | 7.28E-12 |
| ENSMUSG00000079037 | *Prnp* | 1.549274 | 8.957976 | 7.77E-11 |
| ENSMUSG00000079293 | *Clec7a* | 3.198116 | 0.821581 | 4.42E-10 |
| ENSMUSG00000018927 | *Ccl6* | 3.39885 | 0.393266 | 1.02E-08 |
| ENSMUSG00000023992 | *Trem2* | 1.354341 | 3.074181 | 2.30E-07 |
| ENSMUSG00000030579 | *Tyrobp* | 2.208173 | 2.011728 | 1.55E-06 |
| ENSMUSG00000022892 | *App* | 1.225 | 10.43842 | 1.83E-06 |
| ENSMUSG00000069516 | *Lyz2* | 2.174822 | 3.03749 | 2.57E-06 |
| ENSMUSG00000004707 | *Ly9* | 2.23793 | 0.262328 | 2.66E-06 |
| ENSMUSG00000036896 | *C1qc* | 1.999691 | 4.110238 | 2.99E-06 |
| ENSMUSG00000040552 | *C3ar1* | 1.680807 | 1.26491 | 3.48E-06 |
| ENSMUSG00000069515 | *Lyz1* | 2.629014 | 1.000814 | 4.05E-06 |
| ENSMUSG00000073418 | *C4b* | 1.42005 | 7.102349 | 1.07E-05 |
| ENSMUSG00000019969 | *Psen1* | 0.848874 | 5.434479 | 1.20E-05 |
| ENSMUSG00000015451 | *C4a* | 1.441767 | 5.741604 | 2.20E-05 |
| ENSMUSG00000027015 | *Cybrd1* | 1.999986 | 1.791556 | 2.69E-05 |
| ENSMUSG00000020932 | *Gfap* | 1.658875 | 8.593936 | 4.30E-05 |

Supplementary Table S2: Top 20 genes differentially expressed between post-mortem MTG tissue from patients with AD and controls. Additionally, *GFAP*, *NEUROD6*, and *SERPING1* fell outside the top 20, but are associated with AD.

| Ensembl ID | Gene symbol | Log2 FC | Log2 CPM | P-Value |
| --- | --- | --- | --- | --- |
| ENSG00000118785 | *SPP1* | 2.904605 | 5.640599 | 1.01E-11 |
| ENSG00000251792 | *RF00019* | 3.395386 | 1.359578 | 7.79E-10 |
| ENSG00000223298 | *RNY3P8* | 2.995519 | 1.329992 | 5.11E-08 |
| ENSG00000199788 | *RNY3P2* | 2.801296 | 1.463447 | 7.94E-08 |
| ENSG00000243498 | *UBA52P5* | 2.348827 | 1.229709 | 3.36E-07 |
| ENSG00000222509 | *RF00019* | 2.27284 | 1.342453 | 5.82E-07 |
| ENSG00000136750 | *GAD2* | -1.31509 | 5.668942 | 7.84E-07 |
| ENSG00000139318 | *DUSP6* | -1.64103 | 3.269535 | 7.88E-07 |
| ENSG00000200428 | *RF00019* | 2.076545 | 1.128705 | 1.12E-06 |
| ENSG00000186081 | *KRT5* | -3.51567 | -0.18249 | 1.60E-06 |
| ENSG00000200118 | *RF00019* | 2.688251 | 1.598126 | 4.15E-06 |
| ENSG00000275830 | *AL355974.2* | 3.200109 | 1.731332 | 1.37E-05 |
| ENSG00000002933 | *TMEM176A* | 1.627847 | 2.520382 | 1.59E-05 |
| ENSG00000173369 | *C1QB* | 1.765277 | 4.586507 | 1.76E-05 |
| ENSG00000026508 | *CD44* | 3.121055 | 4.077382 | 2.29E-05 |
| ENSG00000163576 | *EFHB* | -3.01175 | 0.806215 | 3.57E-05 |
| ENSG00000183196 | *CHST6* | 1.963519 | 3.333099 | 3.59E-05 |
| ENSG00000122863 | *CHST3* | 1.816107 | 3.331276 | 4.06E-05 |
| ENSG00000200252 | *RF00019* | 3.146761 | 1.301928 | 4.34E-05 |
| ENSG00000188848 | *BEND4* | -1.50046 | 2.028734 | 5.10E-05 |
|  |  |  |  |  |
| ENSG00000131095 | *GFAP* | 1.478094 | 10.18736 | 0.012533 |
| ENSG00000164600 | *NEUROD6* | -1.23075 | 3.532643 | 0.02309 |
| ENSG00000149131 | *SERPING1* | 1.0231 | 4.327277 | 0.023527 |

Supplementary Table S3: Differentially expressed miRNA between hippocampal tissue from APP/PS1 mice and wild-type littermates at 15 months.

| MicroRNA | Log2 FC | Log2 CPM | P-Value |
| --- | --- | --- | --- |
| *mmu-miR-7116-5p* | -1.77659766 | 11.8667953 | 4.70E-10 |
| *mmu-miR-204-5p* | -1.78682701 | 10.62076 | 1.37E-06 |
| *mmu-miR-335-5p* | 1.6810695 | 9.65068644 | 3.72E-06 |
| *mmu-miR-3535* | -1.01790812 | 13.5471796 | 5.80E-06 |
| *mmu-miR-5121* | -0.89305181 | 13.8960423 | 5.04E-05 |
| *mmu-miR-5099* | -0.86267937 | 13.2408062 | 0.0002059 |
| *mmu-miR-128-3p* | -0.73665073 | 15.2680783 | 0.00043643 |
| *mmu-miR-138-5p* | -0.73084807 | 14.8789081 | 0.00046547 |
| *mmu-miR-29a-3p* | -0.72510034 | 14.3877736 | 0.00064187 |
| *mmu-miR-434-3p* | -1.05958494 | 11.0008079 | 0.00080349 |
| *mmu-miR-101b-3p* | 0.7867736 | 10.8017299 | 0.00136466 |
| *mmu-miR-19b-3p* | 0.9561989 | 11.3025501 | 0.0016131 |
| *mmu-miR-381-3p* | 1.3195373 | 9.76662436 | 0.00240092 |
| *mmu-miR-330-3p* | 0.9871243 | 9.49113829 | 0.00523948 |
| *mmu-miR-26a-5p* | 0.5778233 | 14.6909266 | 0.00678561 |
| *mmu-miR-146a-5p* | 0.9840223 | 9.38564529 | 0.00679035 |
| *mmu-miR-29c-3p* | 0.6788836 | 11.363049 | 0.0125626 |
| *mmu-miR-29b-3p* | 0.5219878 | 15.2886921 | 0.01318671 |
| *mmu-miR-219a-5p* | -0.90601545 | 10.4697386 | 0.01357484 |
| *mmu-miR-677-5p* | 0.6356395 | 10.8025264 | 0.01730882 |
| *mmu-miR-329-3p* | -1.66387009 | 9.671779 | 0.01828234 |
| *mmu-miR-7a-5p* | -0.53366947 | 13.1082965 | 0.02119789 |
| *mmu-miR-300-3p* | 0.774125 | 10.6238473 | 0.03086036 |
| *mmu-miR-26b-5p* | 0.793632 | 9.67191044 | 0.03867215 |
| *mmu-miR-7b-5p* | -0.55246216 | 10.9682307 | 0.03908574 |
| *mmu-miR-222-3p* | 0.5537042 | 11.778115 | 0.04146318 |

Supplementary Table S4: Differentially expressed miRNA between post-mortem MTG tissue from patients with Alzheimer’s disease and controls.

| MicroRNA | Log2 FC | Log2 CPM | P-Value |
| --- | --- | --- | --- |
| *hsa-miR-129-5p* | -1.13988462 | 8.57694881 | 0.00556053 |
| *hsa-miR-28-5p* | -1.36421231 | 7.64672392 | 0.00674607 |
| *hsa-miR-151b* | -1.28253881 | 12.0743961 | 0.01184972 |
| *hsa-miR-4484* | 1.8350534 | 9.50843194 | 0.03801881 |
